# Prognostic Value of Eight-Gene Signature in Head and Neck Squamous Carcinoma

**DOI:** 10.3389/fonc.2021.657002

**Published:** 2021-06-18

**Authors:** Baoling Liu, Quanping Su, Jianhua Ma, Cheng Chen, Lijuan Wang, Fengyuan Che, Xueyuan Heng

**Affiliations:** ^1^ Central Laboratory, Linyi People’s Hospital, Linyi, China; ^2^ Linyi Key Laboratory of Tumor Biology, Linyi People’s Hospital, Linyi, China; ^3^ Department of Radiotherapy, NO2 People’s Hospital of Lianyungang, Lianyungang, China; ^4^ Linyi Key Laboratory of Neurophysiology, Linyi People’s Hospital, Linyi, China; ^5^ Department of Neurosurgery, Linyi People’s Hospital, Linyi, China

**Keywords:** head and neck squamous carcinoma, Cox regression, GEO, LASSO, prognostic signature, TCGA

## Abstract

Head and neck cancer (HNC) is the fifth most common cancer worldwide. In this study, we performed an integrative analysis of the discovery set and established an eight-gene signature for the prediction of prognosis in patients with head and neck squamous cell carcinoma (HNSCC). Univariate Cox analysis was used to identify prognosis-related genes (with P < 0.05) in the GSE41613, GSE65858, and TCGA-HNSC RNA-Seq datasets after data collection. We performed LASSO Cox regression analysis and identified eight genes (CBX3, GNA12, P4HA1, PLAU, PPL, RAB25, EPHX3, and HLF) with non-zero regression coefficients in TCGA-HNSC datasets. Survival analysis revealed that the overall survival (OS) of GSE41613 and GSE65858 datasets and the progression-free survival(DFS)of GSE27020 and GSE42743 datasets in the low-risk group exhibited better survival outcomes compared with the high-risk group. To verify that the eight-mRNA prognostic model was independent of other clinical features, KM survival analysis of the specific subtypes with different clinical characteristics was performed. Univariate and multivariate Cox regression analyses were used to identify three independent prognostic factors to construct a prognostic nomogram. Finally, the GSVA algorithm identified six pathways that were activated in the intersection of the TCGA-HNSC, GSE65858, and GSE41613 datasets, including early estrogen response, cholesterol homeostasis, oxidative phosphorylation, fatty acid metabolism, bile acid metabolism, and Kras signaling. However, the epithelial–mesenchymal transition pathway was inhibited at the intersection of the three datasets. In conclusion, the eight-gene prognostic signature proved to be a useful tool in the prognostic evaluation and facilitate personalized treatment of HNSCC patients.

## Introduction

Head and neck cancer (HNC) includes cancers occurring in the tongue, oral cavity, nasopharynx, oropharynx, larynx, and hypopharynx (1). HNC is one of the most common cancers globally, and in the U.S, 38,380 new oral cavity and pharynx cancer cases were reported in 2020 (2). About 90% of HNCs are classified as head and neck squamous cell carcinoma (HNSCC) (3). The most common risk factors for HNSCC include genetic factors, tobacco, and alcohol consumption, and viral infection such as Epstein–Barr virus (EBV) and the human papillomavirus (HPV) (4, 5). Besides, the complexity of its etiology leads to the heterogeneity of HNSCC. HNSCC patients usually present with locally advanced stage and a substantial proportion of them undergo primary surgery (6, 7). Despite the availability of combined modality treatment such as surgery, chemotherapy, radiotherapy, and molecular targeted therapies in the treatment of HNSCC patients with metastatic and/or recurrent HNSCC, HNSCC is reported to have a poor prognosis (8). Cancer prevention is an important component of HNSCC than treatment. Nowadays, safe and efficacious HPV vaccines against the HPV types that cause 70% of cervical cancer and other HPV-related cancers or diseases have been introduced in 99 countries and territories (9). Lifestyle interventions are known to be important components of cancer prevention. However, in the post-genomic era, a better understanding of the genetic factors and other associated prevention strategies of HNSCC remains essential.

Recent advances in sequencing technologies especially the “second-generation” sequencing technologies have allowed in-depth molecular characterization, stratification of cancer patients, and development of individualized accurate treatment strategies based on specific markers (10, 11). Besides, the rapid development of computer technology has also facilitated the development of bioinformatic tools. Based on enormous amounts of biological data and advanced bioinformatic tools, several studies have explored and developed tumor models (12). Among these, deep learning-based survival prediction has been used to guide clinicians in predicting prognosis and devising individualized treatment strategies (13). To preoperatively identify occult peritoneal metastasis in gastric cancer, an individualized nomogram was developed and validated (14). Sanghani et al. developed and validated a nomogram that could predict tumor recurrence in breast cancer patients using two independent population-based datasets (15). Nassiri et al. developed a prediction model of early recurrence risk combining clinical and molecular factors in meningioma (16). Besides, several prognostic prediction models for different cancers have been constructed based on prognosis-related molecular features (17, 18). A robust miRNA-based signature for predicting the prognostic outcome of HNSCC with high accuracy has been developed (19). However, additional gene signatures and more accurate models are needed for predicting the prognosis and guiding therapeutic decision-making.

In this study, we performed an integrative analysis to establish an eight-gene signature for the prediction of prognosis in patients with head and neck squamous cell carcinoma (HNSCC). LASSO Cox regression analysis identified eight genes (CBX3, GNA12, P4HA1, PLAU, PPL, RAB25, EPHX3, and HLF) with non-zero regression coefficients in TCGA-HNSC datasets. The nomogram was constructed, and GSVA revealed the associated functional signaling pathways.

## Materials and Methods

### Data Collection

We downloaded mRNA sequencing data and clinical information for HNSCC patients from The Cancer Genome Atlas (TCGA) database as the training dataset (https://portal.gdc.cancer.gov/). Gene expression profiles and clinical information of HNSCC patients in the GSE23036, GSE41613, GSE65858, GSE27020, and GSE42743 datasets were downloaded from Gene Expression Omnibus (GEO) database (https://www.ncbi.nlm.nih.gov/geo/) as the test datasets. **Table 1** presents detailed information of the test datasets based on tumor type, sample, and platform.

**Table 1:** Detailed information of the test datasets on tumor type, sample, and platform.

GEO number	Tumor type	Sample	Platform
GSE65858	HNSCC	270	GPL10558 Illumina HumanHT-12 V4.0 expression beadchip
GSE41613	OSCC	97	GPL570 Affymetrix Human Genome U133 Plus 2.0 Array
GSE27020	laryngeal cancer	109	GPL96 [HG-U133A] Affymetrix Human Genome U133A Array
GSE42743	OSCC	103	GPL570 Affymetrix Human Genome U133 Plus 2.0 Array

### Identification of Prognostic Gene Signature

The mRNA sequencing data and clinical information from TCGA, GSE41613, and GSE65858 were analyzed by univariate Cox analysis in the “survival” R package to identify genes associated with prognosis. Intersecting genes were identified as prognosis-related genes. To further screen for potential prognostic biomarkers, gene expression profiling interactive analysis (GEPIA, http://gepia.cancer-pku.cn) and gene expression profiling of the GSE23036 dataset were used for differential expression analysis. GEPIA is an online data tool that uses a standard processing pipeline to analyze RNA-seq data of 9,736 tumors and 8,587 normal samples from the TCGA and Genotype-Tissue Expression (GTEx) database (20).

### Construction of a Prognostic mRNA Signature

In this study, we included 499 samples with complete clinical survival information from the TCGA-HNSC database. The ‘glmnet’ R package in R was used to perform LASSO Cox regression analysis (least absolute shrinkage and selection operator, LASSO). The time-dependent receiver operating characteristic (ROC) curve was plotted to evaluate the predictive accuracy of the mRNA signature. The area under the ROC curve (AUC) of 1, 3, and 5 years was calculated by the ‘survival ROC’ R package.

### Prognostic Significance of the Eight-mRNA Prognostic Signature in Independent Validation Datasets

The prognostic value of the prognostic mRNA signature in predicting OS and DFS was confirmed in the GSE41613 and GSE65858, and GSE27020 and GSE42743 datasets, respectively. Time-dependent ROC curve analysis was also plotted to prove the survival prediction accuracy.

### The Prognostic Value of the Eight-mRNA Prognostic Model Independent of Other Clinical Features

To confirm the relationship between the prognostic model and different clinical features, including TNM stage, grade, age, gender, T stage, N stage, and M stage, we randomly divided the TCGA-HNSC samples into two groups. Patients were separately classified into stage I/II and III/IV subgroups, grade I/II and III/IV subgroups, age <65 and age ≥65 subgroups, male and female subgroups, T0–T2 and T3/4 subgroups, N0 and N + subgroups, and M0 and M1 + Mx subgroups. KM survival analysis of the specific subtypes of different clinical characteristics was performed to confirm the independent prognostic value of the eight-mRNA prognostic signature.

### Construction and Validation of the Prediction Nomogram

A nomogram constructed using several independent indicators can be used for multiple predictions (21–23). Univariate and multivariate Cox regression analyses were used to screen for independent risk factors, which were used to construct a nomogram for the prediction of HNC prognosis. Forest plots were used to display the results of univariate and multivariate Cox regression analyses. Three independent prognostic factors, M stage, N stage, and the eight-mRNA gene signature, were used to construct the nomogram. The ‘regplot’ package in R was used to build the nomogram. To further assess the discrimination power and accuracy of the nomogram, calibration curves were used to predict the OS at 3 and 5 years. Besides, decision curve analysis (DCA) was performed to evaluate the clinical utility of the nomogram by calculating the clinical net benefit across the range of decision threshold probabilities in the TCGA-HNSC dataset.

### Gene Set Variation Analysis

Here, samples from the TCGA-HNSC, GSE65858, and GSE41613 datasets were divided into high and low-risk groups based on the risk score. The “GSVA” package in R was used to perform GSVA between the high-risk and low-risk groups, using the hallmark gene sets as a reference. We set |log2FC| >2 and P <0.05 as the cut-off values to identify valuable signaling pathways. The intersection between integrated differential pathways from the three datasets was determined to identify the activated and suppressed pathways.

### Survival Analysis

To determine if there were significant differences in survival between the high and low-risk groups, Kaplan–Meier survival curves were plotted. The ‘survival’ package in R was used to perform a two-sided log-rank test and univariate and multivariate Cox regression analyses.

## Results

### Identification of Prognostic Candidate Genes

**Figure 1:**
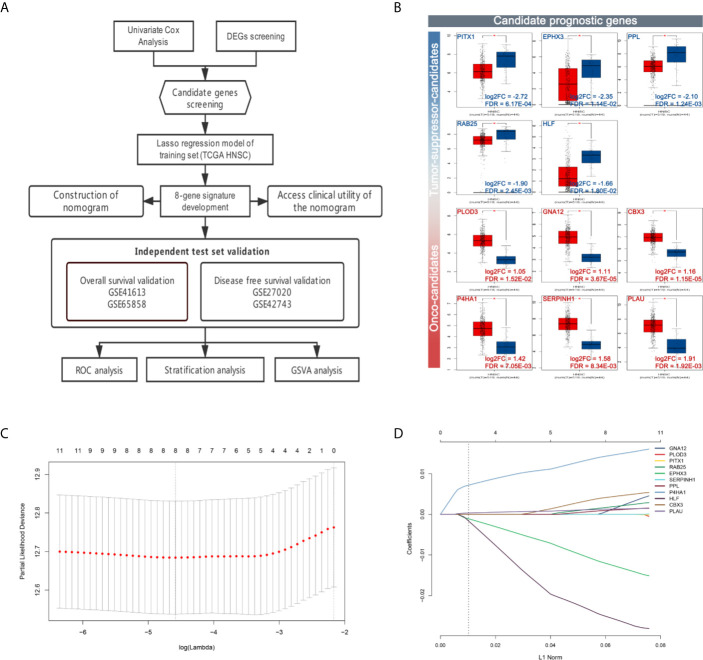
Workflow and construction of LASSO Cox regression model. **(A)** Work flow of the study. **(B)** Expression of the candidate prognostic genes. **(C)** The LASSO coefficients profiles of 11 candidate prognostic genes. **(D)** Tuning parameter (*λ*) selection cross-validation error curve. The vertical lines were drawn at the optimal values by the minimum criteria and the 1-SE criteria. We choose the right line by 1-SE criteria where the eight-gene signature was selected.

The flow chart of data preparation, processing, analysis, and validation is shown in **Figure 1A**. The GSE41613 and GSE65858 datasets (Platforms: GPL570 and GPL10558) were downloaded from the GEO database (https://www.ncbi.nlm.nih.gov/geo/), including the expression profile and clinical information of 97 HPV-negative OSCC patients and 270 head and neck squamous cell carcinoma arrays, respectively. The TCGA RNA-seq dataset and clinical characteristics of 528 HNSC patients were downloaded from The Cancer Genome Atlas data portal (TCGA, https://portal.gdc.cancer.gov/repository). Univariate Cox regression analysis was performed, and prognosis-related genes were identified (with P < 0.05) from the GSE41613, GSE65858, and TCGA-HNSC RNA-Seq datasets. Genes associated with prognosis at the intersection of all the three datasets and differentially expressed genes of HNSC (with |log2FC| > 1 and FDR < 0.05) from GEPIA were screened as candidate prognostic genes. A total of eleven hub genes were identified; *PITX1, EPHX3, PPL, RAB25*, and *HLF* were found to be down-regulated, and PLOD3, *GNA12, CBX3, P4HA1, SERPINH1*, and *PLAU* were up-regulated (**Figure 1B**).

### Development of an Eight-mRNA Prognostic Signature

**Figure 2:**
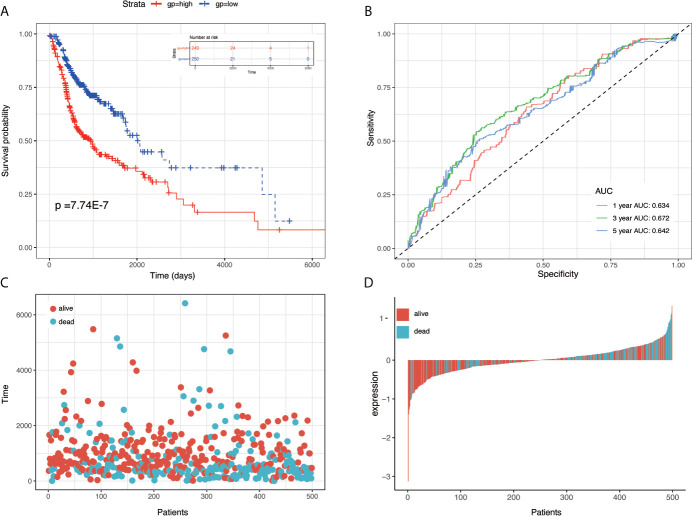
KM survival, risk score by eight-gene signature and time-dependent ROC curves in the TCGA-HNSC training set. **(A)** KM survival analysis between high- and low-risk samples in TCGA-HNSC. **(B)** Time-dependent ROC curve for OS of TCGA-HNSC; the AUC was assessed at 1, 3, and 5 years. **(C)** Relationship between survival time (day) and risk score rank. **(D)** Relationship between survival status and risk score rank.

To establish the prognostic signature, LASSO Cox regression analysis was performed, and eight genes were identified (CBX3, GNA12, P4HA1, PLAU, PPL, RAB25, EPHX3, and HLF) with non-zero regression coefficients in TCGA-HNSC datasets(**Figures 1C, D**). The risk score for each case was calculated using the following formula. (Risk score = CBX3 expression * 4.293E-03 + GNA12 expression * 1.124E-03 + P4HA1 expression * 1.450E-02 + PLAU expression * 1.283E-03 + PPL expression * 1.023E-03 + RAB25 expression * 1.824E-03 + EPHX3 expression *(1.243E-02)+ HLF expression *(−2.550E-02)). A total of 499 patients in TCGA-HNSC were divided into high-risk group and low risk group. Kaplan–Meier survival curve demonstrated that patients with a high-risk score had worse prognosis compared with the low risk score group (P < 0.0001, p = 7.74E-07) (**Figures 2A, C, D**). Time-dependent ROC was used to assess the prognostic value of the eight-mRNA prognostic signature. The AUCs of the 1-,3-, and 5-year risk scores were 0.634, 0.672, 0.764, and 0.642, respectively (**Figure 2B**).

### Validation of the Eight-mRNA Prognostic Signature

**Figure 3:**
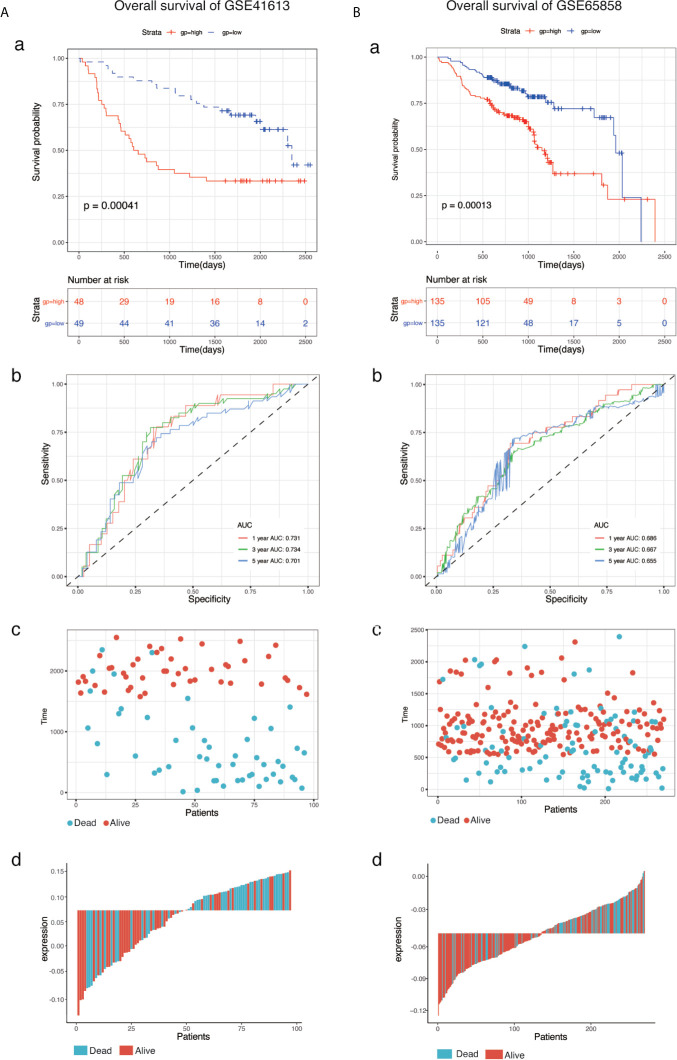
KM survival, risk score by eight-gene signature and time-dependent ROC curves in the OS validation datasets. **(A)** GSE41613, **(B)** GSE65858. (a) KM survival analysis between high- and low-risk samples. (b) Time-dependent ROC curve for overall survival of validation datasets, the AUC was assessed at 1, 3, and 5-years. (c) Relationship between survival time (day) and risk score rank. (d) Relationship between survival status and risk score rank.

**Figure 4:**
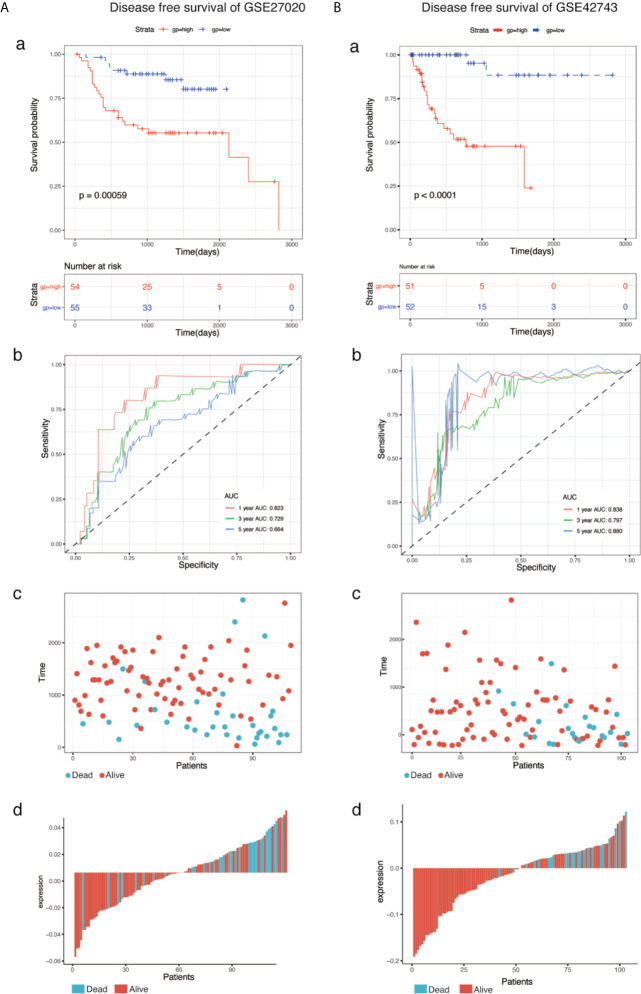
KM survival, risk score by eight-gene signature and time-dependent ROC curves in the DFS validation datasets. **(A)** GSE27020, **(B)** GSE42743. (a) KM survival analysis between high and low risk samples. (b) Time-dependent ROC curve for overall survival of validation datasets, the AUC was assessed at 1-, 3- and 5-years. (c) Relationship between survival time (day) and risk score rank. (d) Relationship between survival status and risk score rank.

To determine the predictive accuracy of the eight-mRNA prognostic signature, two datasets (GSE41613and GSE65858) were used for OS validation, and another two external datasets (GSE27020 and GSE42743) were used for DFS validation. Patients in the validation datasets were divided into the high-risk or low-risk groups using the median risk score as the cut-off. Results from survival analysis showed that both the overall survival (OS) of the GSE41613 and GSE65858 datasets and the progression-free survival(DFS)of the GSE27020 and GSE42743 datasets in the low-risk group, exhibited a better trend in survival compared with the high-risk group. The prognostic value in the OS validation dataset was evaluated in the GSE41613(p = 0.00041)and GSE65858(p = 0.00013) datasets. The AUC for 1-, 3- and 5-year survival was 0.731, 0.734, 0.701 and 0.686, 0.667, 0.655, for GSE41613 and GSE65858, respectively (**Figure 3**). A similar trend in survival in the DFS validation datasets, GSE27020(p = 0.00059)and GSE42743(p <0.0001). The AUC for 1-, 3- and 5-year survival was 0.823, 0.729, 0.664 and 0.838, 0.797, 0.880, for GSE41613 and GSE65858, respectively (**Figure 4**). These results indicated that the established eight-mRNA signature could effectively predict the prognosis of HNSC patients.

### The Prognostic Value of theEight-mRNA Signature

**Figure 5:**
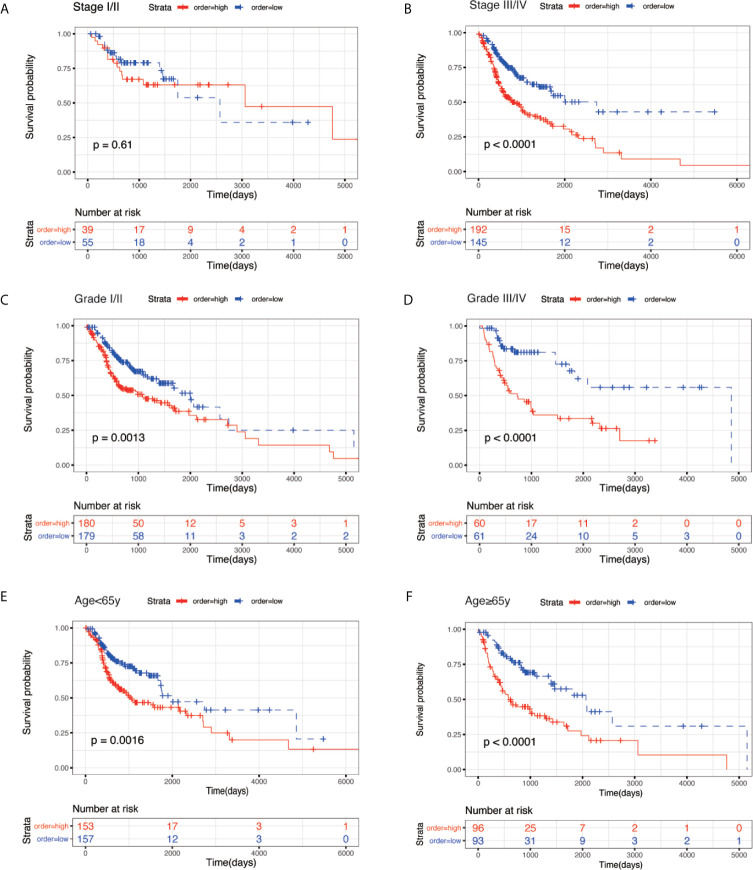
KM survival subgroup analysis for all patients with HNSC according to the eight-gene signature stratified by clinical characteristics. **(A)** Stage I/II. **(B)** Stage III/IV. **(C)** Grade I/II. **(D)** Grade III/IV. **(E)** age <65 year. **(F)** age ≥ 65 year.

**Figure 6:**
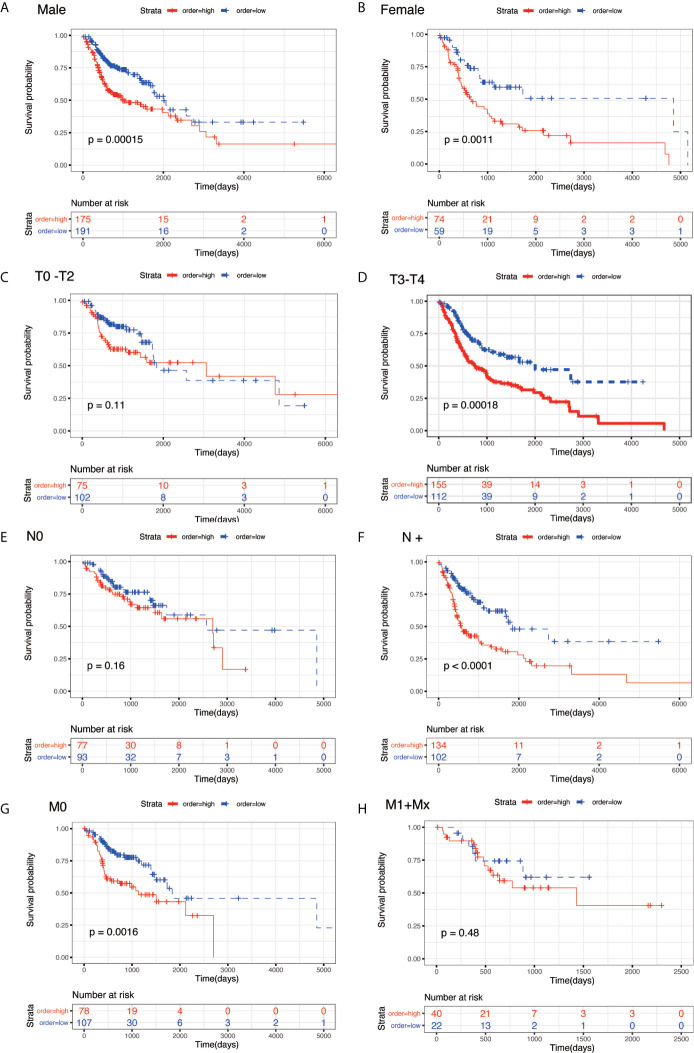
KM survival subgroup analysis for all patients with HNSC according to the eight-gene signature stratified by clinical characteristics. **(A)** male. **(B)** female. **(C)** T0–T2. **(D)** T3/T4. **(E)** N0. **(F)** N+. **(G)** M0. **(H)** M1 + Mx**.

To evaluate the prognostic significance of the model in different clinicopathological features, all clinical variables, samples were randomly divided into two subgroups based on TNM stage, grade, age, gender, pathological T stage, pathological N stage, and pathological M stage. Patients in different subgroups were further divided into the high-risk and low-risk groups, using the median risk score of the prognostic model as the cut-off value. As shown in **Figures 5** and **6**, KM survival analysis of different subgroups indicated that in 12 subgroups, including the stage III/IV, grade I/II, grade III/IV, age <65 y, age ≥65 y, male, female, T3/4 stage, N+ stage, and M0 stage, the prognostic model significantly correlated with the survival outcomes of HNSC patients. However, in the regional-stage subgroups, including stage and N0, the eight-mRNA prognostic model failed to show any positive prognostic value (**Figures 5** and **6**).

### The Establishment and Clinical Application of the Nomogram

**Figure 7:**
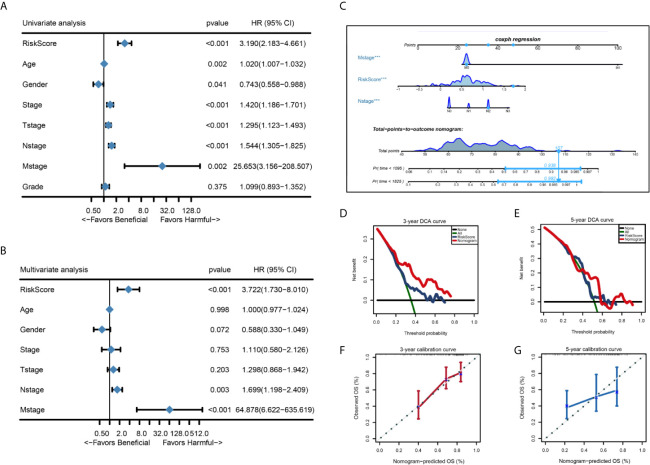
Forest plot summary of univariate and multivariate analysis of 8-gene signature and nomogram to predict 3- and 5-year OS in TCGA-HNSC training set. **(A, B)** Univariable and multivariable analysis of OS for the TCGA-HNSC patients. The blue diamond squares on the transverse lines represent the HR and the gray transverse lines represent 95% CI. And the p value and 95% CI for each clinicopathological character were displayed in detail. **(C)** The nomogram for predicting proportion of patients with 3- or 5 - year OS. **(D, E)** Calibration curve for the prediction of 3- or 5- year overall survival. **(F, G)** DCA curve for the prediction of 3- or 5- year overall survival.

We performed univariate Cox regression analysis and multivariate Cox regression analysis to identify independent risk factors associated with the prognosis of HNSC. Patients from the TCGA-THCA dataset with complete clinical data including age, gender, stage, T stage, N stage, M stage, grade, and the risk score were included in the identification of prognostic factors. Results from the univariate and multivariate analysis showed that risk score of the eight-mRNA prognostic signature [univariate analysis: P < 0.001, HR = 3.190, 95% CI: 2.183–4.661; multivariate analysis: P < 0.001, HR = 3.722, 95%CI: 1.730–8.010)], N stage (univariate analysis: P < 0.001, HR = 1.544, 95%CI: 1.305–1.825; multivariate analysis: P = 0.003, HR = 1.699, 95%CI: 1.198–2.409) and M stage (univariate analysis: P = 0.002, HR = 25.653, 95%CI: 3.156–208.507; multivariate analysis: P < 0.001, HR = 64.878, 95%CI: 6.622–635.619) were significant independent factors (**Figures 7A, B**). Therefore, the three independent prognostic factors were selected and used to construct a nomogram for predicting the probability of 3- and 5-year OS in HNSC patients (**Figure 7C**). Each prognostic parameter had a score, and the sum of the three prognostic parameter scores was used to predict the 3-year and 5-year OS. The higher the total score, the worse the prognosis. Besides, to determine the clinical utility of the nomogram, DCA was used to compare the net benefits of different models, including none, all, risk score, and nomogram. As shown in **Figures 7D, E**, compared with the other three groups, the nomogram revealed higher net benefits with wider threshold probabilities. The DCA results also indicated that the nomogram had better clinical benefit than the risk score calculated based on the eight-mRNA gene signature alone. Moreover, the 3- and 5-year OS calibration curves for the TCGA-HNSC dataset showed similar performance with the ideal model, indicating that the nomogram has good predictive discrimination power and accuracy (**Figures 7F, G**).

### GSVA Analysis

**Figure 8:**
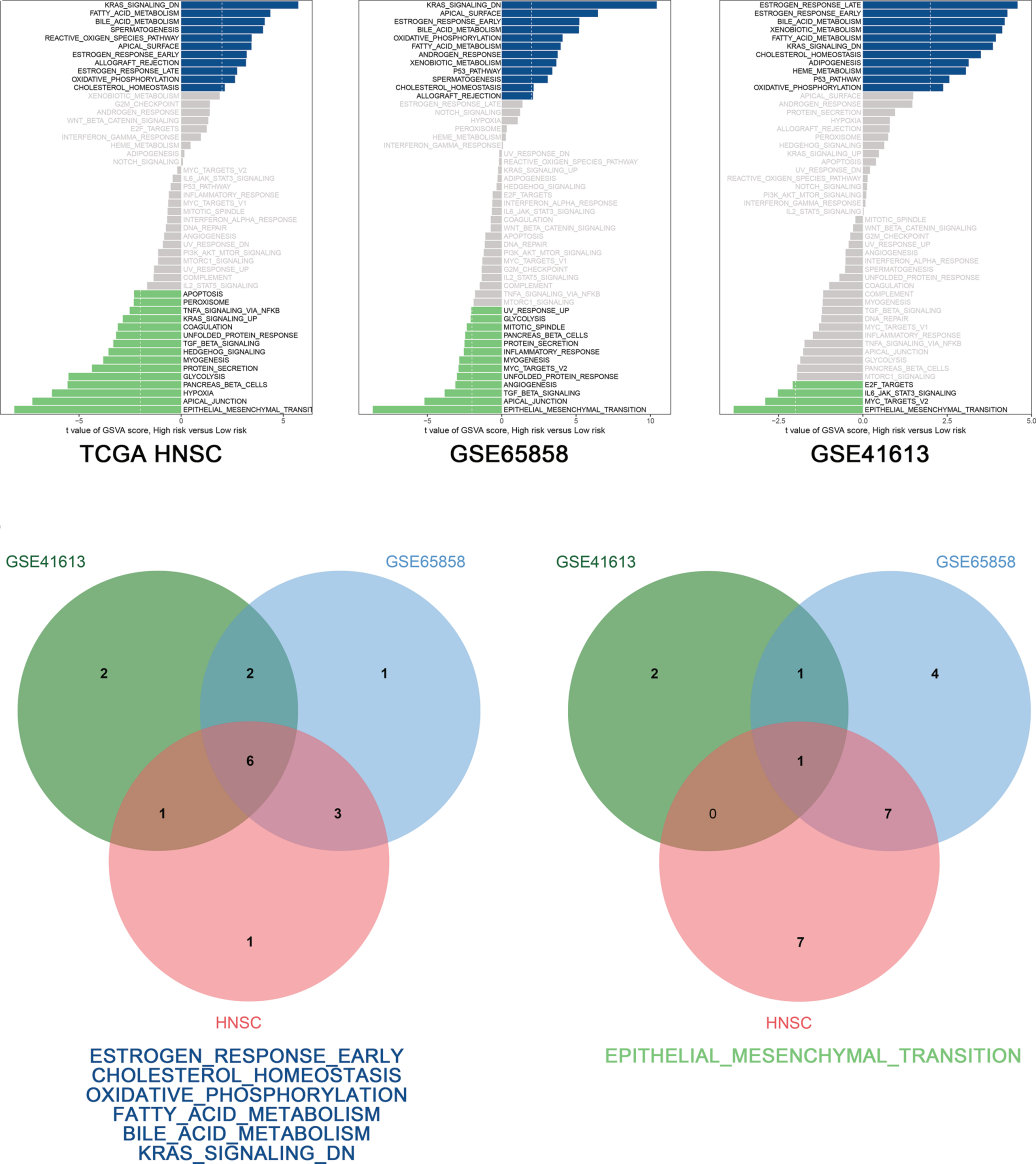
GSVA analysis. **(A)** GSVA of the TCGA-HNSC (a), GSE65858 (b), GSE41613 (c) data sets. **(B)** Venn diagram of the activated (a) and suppressed (b) gene sets in the indicated data sets.

To explore the biological function of the prognostic signature between the high-risk and low-risk group, we performed GSVA *via* “GSVA” package in R and using hallmark gene sets as reference. According to the cut-off criteria (|log2FC| > 2 and P < 0.05), 11 pathways were found to be activated and 15 pathways inhibited in the TCGA-HNSC dataset, 12 pathways were activated and 13 pathways inhibited in the GSE65858 dataset, and 11 pathways were activated and four pathways were inhibited in the GSE41613 dataset (**Figure 8Aa–c**). **Figure 8** shows that six pathways were activated in the intersection of the TCGA-HNSC, GSE65858, and GSE41613 datasets, including estrogen response early, cholesterol homeostasis, oxidative phosphorylation, fatty acid metabolism, bile acid metabolism, and Kras signaling. The epithelial–mesenchymal transition pathway was inhibited at the intersection of the three datasets (**Figure 8Bb**).

## Discussion

Genomic studies of HNSCC have greatly increased our understanding of genetic heterogeneity, disease diversity, and key genes driving tumorigenesis (24). With recent developments in sequencing techniques, such as high-throughput sequencing technologies (25), the emergence of enormous amounts of data has led to a wide variety of methods and tools for data analysis. Based on gene expression profiles and clinical data from public databases, a variety of prognostic models have been constructed. Genes that could serve as therapeutic targets and prognostic biomarkers for head and neck cancer were identified by Fan et al. (26). Li et al. performed a systematic analysis of the immunogenomic landscape and identified IRGs as potential biomarkers of HNSCC (27). Th identified molecules and constructed models can also provide an immeasurable reference for further basic and clinical research.

In this study, differentially expressed genes between the tumor and normal tissues were identified, indicating that multiple genetic abnormalities might be involved in HNSCC tumorigenesis. Univariate Cox analysis and LASSO Cox regression analysis identified eight (CBX3, GNA12, P4HA1, PLAU, PPL, RAB25, EPHX3, and HLF) prognosis-related genes. CBX3 promotes cell proliferation and predicts poor prognosis in glioma, and P4HA1 is a biomarker of unfavorable prognosis in malignant melanomas (28, 29). Aberrant expression of RAB25 promotes tumorigenesis of skin squamous cell carcinoma (30), while HLF down-regulation promotes distant metastases in non-small cell lung cancer (31). We constructed the eight-gene prognostic model and calculated the risk score based on the model. Our results revealed that patients with a high-risk score had a worse prognosis than those with a low-risk score. To further assess the predictive accuracy of the model, two datasets were used for OS and DFS validation, respectively. Our results showed that based on the OS and DFS, the low-risk group exhibited a better trend in survival, compared with the high-risk group. Subgroup analysis also revealed that the prognostic model was significantly correlated with the survival of HNSC patients based on 12 subgroups. Our results were consistent with previous studies on CBX3, P4HA1, and RAB25. Limited studies on the function of GNA12, PLAU, PPL, EPHX3, and HLF are available. Our results might serve as a basis for future studies of these genes.

Univariate and multivariate Cox regression analyses were performed to screen for independent prognostic factors, and construct a nomogram. Each prognostic parameter had a score, and the sum of the three prognostic parameter scores was used to predict the 3- and 5-year OS. The easy-to-use nomogram has its unique advantages in clinical practice compared with the AJCC staging system (32). A prognostic nomogram for patients with newly diagnosed lower-grade gliomas was constructed and validated in a large-scale Asian cohort and a free online tool for this nomogram is available for use in clinical practice (33). To identify occult peritoneal metastasis in patients with advanced gastric cancer, Han et al. developed and validated an individualized nomogram (14). There are several studies reporting nomogram construction and validation in various cancers, including gallbladder, prostate, and breast cancers (34–36). However, studies on head and neck cancer are not available.

GSAV was performed to gain in-depth insights into the molecular functions of the eight-mRNA signature. A total of six pathways were found to be activated in the intersection of the TCGA-HNSC, GSE65858, and GSE41613 datasets, including early estrogen response, cholesterol homeostasis, oxidative phosphorylation, fatty acid metabolism, bile acid metabolism, and Kras signaling. The epithelial–mesenchymal transition pathway was found to be inhibited in the intersection of the three datasets. The estrogen receptor is closely related to breast cancer (37), and estrogen can significantly promote tumor growth by combining with the estrogen receptor (38). In another study, the cholesterol transporter links cholesterol homeostasis and tumor immunity (39). As one of the cancer signals, oxidative phosphorylation supports the development of a variety of cancers (40, 41). Cancer cells have characteristic alterations in metabolisms, such as fatty acid metabolism and bile acid metabolism. Therefore, limiting the availability of fatty acids can control cancer cell proliferation (42). Kras signaling is essential for tumorigenesis and specific targeting of tumours using mutant KRAS has been used in clinical practice (43). Our results showed that the prognostic model is associated with the activation of the identified six pathways, which is consistent with previous findings. The phenomenon of epithelial–mesenchymal transition refers to when epithelial cells loosen cell-cell adhesion structures and promote tumor progression (44). Numerous studies have shown that epithelial–mesenchymal transition plays important roles in tumor initiation and malignant progression (45, 46). Our eight-mRNA signature was reported to be associated with poor prognosis in HNSCC patients, which is also consistent with the inhibition of EMT.

This study also has several limitations. First, in our current study, we presented bioinformatic evidence suggesting that the eight-mRNA signature can accurately predict the prognosis of HNSCC and constructed a nomogram. Data were generated from public databases, which lack experimental validation. Secondly, a prognostic model consisting of eight mRNAs was constructed and validated in our study. Several studies have investigated the functions of CBX3, P4HA1, and RAB25, but few studies on the function of GNA12, PLAU, PPL, EPHX3, and HLF are available. Therefore, more studies on the functions of these genes are needed in the future. Thirdly, to our knowledge, viruses have been implicated in head and neck cancers (47). In this study, we analyzed the correlations between the prognostic model and common clinical features, including TNM stage, grade, age, gender, pathological T stage, pathological N stage, and pathological M stage. However, EBV or HPV status was not included in the analysis and which is also very important when stratifying prognosis and in clinical decision making. Therefore, there is a need to explore the correlation between virus status and the 8‐mRNA signature in HNSCC in the future.

## Conclusion

A better understanding of the genetic factors and models for predicting prognosis and personalizing therapeutic decision-making in HNSCC is needed. In this study, LASSO Cox regression analysis is conducted to identify the eight-mRNA signature (CBX3, GNA12, P4HA1, PLAU, PPL, RAB25, EPHX3, and HLF) predicting the prognosis of HNSCC. Then, the constructed nomogram has been identified clinical utility. GSVA results show that six pathways are activated, while the epithelial–mesenchymal transition pathway is inhibited in our gene signature.

## Data Availability Statement

Publicly available datasets were analyzed in this study. This data can be found here: https://portal.gdc.cancer.gov/; https://www.ncbi.nlm.nih.gov/geo/.

## Author Contributions

BL conducted the experiments, wrote, and edited the manuscript; QS, JM, and CC reviewed and edited the manuscript; LW and FC contributed to analysis of the data and drafted the manuscript. XH contributed to the conception, design, and analysis of the manuscript. All authors contributed to the article and approved the submitted version.

## Funding

This work was supported by the Nature Science Foundation of Bengbu Medical College (BYKY18177) and Postdoctoral Innovation Project of Shandong Province in 2020 (no.20203068).

## Conflict of Interest

The authors declare that the research was conducted in the absence of any commercial or financial relationships that could be construed as a potential conflict of interest.

## Abbreviations

HNC, head and neck cancer; HNSCC, head and neck squamous cell carcinoma; TCGA, The Cancer Genome Atlas; GEO, Gene Expression Omnibus; LASSO, least absolute shrinkage and selection operator; ROC, receiver operating characteristic curve analysis; DFS, progression-free survival; OS, overall survival.

## References

[1] Rasmussen JH Lelkaitis G Hakansson K Vogelius IR Johannesen HH Fischer BM. Intratumor Heterogeneity of PD-L1 Expression in Head and Neck Squamous Cell Carcinoma. Br J Cancer (2019) 120(10):1003–6. 10.1038/s41416-019-0449-y PMC6734649 30967647

[2] Siegel RL Miller KD Jemal A. Cancer Statistics, 2020. CA Cancer J Clin (2020) 70(1):7–30. 10.3322/caac.21590 31912902

[3] Ma H Chang H Yang W Lu Y Hu J Jin S. A Novel IFNalpha-induced Long Noncoding RNA Negatively Regulates Immunosuppression by Interrupting H3K27 Acetylation in Head and Neck Squamous Cell Carcinoma. Mol Cancer (2020) 19(1):4. 10.1186/s12943-019-1123-y 31907020 PMC6943933

[4] Zhong Q Fang J Huang Z Yang Y Lian M Liu H. A Response Prediction Model for Taxane, Cisplatin, and 5-Fluorouracil Chemotherapy in Hypopharyngeal Carcinoma. Sci Rep (2018) 8(1):12675. 10.1038/s41598-018-31027-y 30139993 PMC6107664

[5] Gu X Wang L Boldrup L Coates PJ Fahraeus R Sgaramella N. Ap001056.1, A Prognosis-Related Enhancer RNA in Squamous Cell Carcinoma of the Head and Neck. Cancers (Basel) (2019) 11(3):347. 10.3390/cancers11030347 PMC6468641 30862109

[6] Tang Q Xie M Yu S Zhou X Xie Y Chen G. Periodic Oxaliplatin Administration in Synergy With PER2-Mediated Pcna Transcription Repression Promotes Chronochemotherapeutic Efficacy of OSCC. Adv Sci (Weinh) (2019) 6(21):1900667. 10.1002/advs.201900667 31728273 PMC6839751

[7] Harari PM Harris J Kies MS Myers JN Jordan RC Gillison ML. Postoperative Chemoradiotherapy and Cetuximab for High-Risk Squamous Cell Carcinoma of the Head and Neck: Radiation Therapy Oncology Group Rtog-0234. J Clin Oncol (2014) 32(23):2486–95. 10.1200/JCO.2013.53.9163 PMC4121506 25002723

[8] Vermorken JB Peyrade F Krauss J Mesia R Remenar E Gauler TC. Cisplatin, 5-Fluorouracil, and Cetuximab (PFE) With or Without Cilengitide in Recurrent/Metastatic Squamous Cell Carcinoma of the Head and Neck: Results of the Randomized Phase I/II ADVANTAGE Trial (Phase II Part). Ann Oncol (2014) 25(3):682–8. 10.1093/annonc/mdu003 PMC3933250 24567516

[9] Drolet M Benard E Perez N Brisson M H. P. V. V. I. S. Group. Population-Level Impact and Herd Effects Following the Introduction of Human Papillomavirus Vaccination Programmes: Updated Systematic Review and Meta-Analysis. Lancet (2019) 394(10197):497–509. 10.1016/S0140-6736(19)30298-3 31255301 PMC7316527

[10] Mateo J Chakravarty D Dienstmann R Jezdic S Gonzalez-Perez A Lopez-Bigas N. A Framework to Rank Genomic Alterations as Targets for Cancer Precision Medicine: The ESMO Scale for Clinical Actionability of Molecular Targets (Escat). Ann Oncol (2018) 29(9):1895–902. 10.1093/annonc/mdy263 PMC6158764 30137196

[11] Johansson HJ Socciarelli F Vacanti NM Haugen MH Zhu Y Siavelis I. Breast Cancer Quantitative Proteome and Proteogenomic Landscape. Nat Commun (2019) 10(1):1600. 10.1038/s41467-019-09018-y 30962452 PMC6453966

[12] Jin L Tao H Karachi A Long Y Hou AY Na M. CXCR1- or CXCR2-modified Car T Cells Co-Opt IL-8 for Maximal Antitumor Efficacy in Solid Tumors. Nat Commun (2019) 10(1):4016. 10.1038/s41467-019-11869-4 31488817 PMC6728370

[13] Kim DW Lee S Kwon S Nam W Cha IH Kim HJ. Deep Learning-Based Survival Prediction of Oral Cancer Patients. Sci Rep (2019) 9(1):6994. 10.1038/s41598-019-43372-7 31061433 PMC6502856

[14] Dong D Tang L Li ZY Fang MJ Gao JB Shan XH. Development and Validation of an Individualized Nomogram to Identify Occult Peritoneal Metastasis in Patients With Advanced Gastric Cancer. Ann Oncol (2019) 30(3):431–8. 10.1093/annonc/mdz001 PMC6442651 30689702

[15] Sanghani M Truong PT Raad RA Niemierko A Lesperance M Olivotto IA. Validation of a Web-Based Predictive Nomogram for Ipsilateral Breast Tumor Recurrence After Breast Conserving Therapy. J Clin Oncol (2010) 28(5):718–22. 10.1200/JCO.2009.22.6662 PMC2834390 20048188

[16] Nassiri F Mamatjan Y Suppiah S Badhiwala JH Mansouri S Karimi S. DNA Methylation Profiling to Predict Recurrence Risk in Meningioma: Development and Validation of a Nomogram to Optimize Clinical Management. Neuro Oncol (2019) 21(7):901–10. 10.1093/neuonc/noz061 PMC6620635 31158293

[17] Cavalieri S Mariani L Vander Poorten V Van Breda L Cau MC Lo Vullo S. Prognostic Nomogram in Patients With Metastatic Adenoid Cystic Carcinoma of the Salivary Glands. Eur J Cancer (2020) 136:35–42. 10.1016/j.ejca.2020.05.013 32629365

[18] Huang X Zhang X Wang X Rong X Li Y Li H. A Nomogram to Predict Symptomatic Epilepsy in Patients With Radiation-Induced Brain Necrosis. Neurology (2020) 95(10):e1392–403. 10.1212/WNL.0000000000010190 32631922

[19] Zhao X Cui L. A Robust six-miRNA Prognostic Signature for Head and Neck Squamous Cell Carcinoma. J Cell Physiol (2020) 235(11):8799–811. 10.1002/jcp.29723 32342519

[20] Tang Z Li C Kang B Gao G Li C Zhang Z. GEPIA: A Web Server for Cancer and Normal Gene Expression Profiling and Interactive Analyses. Nucleic Acids Res (2017) 45:W98–W102. 10.1093/nar/gkx247 28407145 PMC5570223

[21] Serenari M Han K Ravaioli F Kim S Cucchetti A Han D. A Nomogram Based on Liver Stiffness Predicts Postoperative Complications in Patients With Hepatocellular Carcinoma. J Hepatol (2020) 73(4):855–62. 10.1016/j.jhep.2020.04.032 32360997

[22] Huo T Liu P Hsu C. Nomogram to Predict Surgical Hepatocellular Carcinoma With Child-Pugh B: Feasibility and Overlooked Predictors. J Hepatol (2020) 72(5):1032–3. 10.1016/j.jhep.2019.12.024 32122721

[23] Lo S Ma J Scolyer R Haydu L Stretch J Saw R. Improved Risk Prediction Calculator for Sentinel Node Positivity in Patients With Melanoma: The Melanoma Institute Australia Nomogram. J Clin Oncol Off J Am Soc Clin Oncol (2020) 38(24):2719–27. 10.1200/jco.19.02362 PMC7430218 32530761

[24] Hammerman PS Hayes DN Grandis JR. Therapeutic Insights From Genomic Studies of Head and Neck Squamous Cell Carcinomas. Cancer Discovery (2015) 5(3):239–44. 10.1158/2159-8290.CD-14-1205 PMC4355279 25643909

[25] Reuter JA Spacek DV Snyder MP. High-Throughput Sequencing Technologies. Mol Cell (2015) 58(4):586–97. 10.1016/j.molcel.2015.05.004 PMC4494749 26000844

[26] Fan G Tu Y Wu N Xiao H. The Expression Profiles and Prognostic Values of HSPs Family Members in Head and Neck Cancer. Cancer Cell Int (2020) 20:220. 10.1186/s12935-020-01296-7 32523426 PMC7278206

[27] Li L Wang XL Lei Q Sun CZ Xi Y Chen R. Comprehensive Immunogenomic Landscape Analysis of Prognosis-Related Genes in Head and Neck Cancer. Sci Rep (2020) 10(1):6395. 10.1038/s41598-020-63148-8 32286381 PMC7156482

[28] Zhao SP Wang F Yang M Wang XY Jin CL Ji QK. CBX3 Promotes Glioma U87 Cell Proliferation and Predicts an Unfavorable Prognosis. J Neurooncol (2019) 145(1):35–48. 10.1007/s11060-019-03286-w 31502042

[29] Eriksson J Le Joncour V Jahkola T Juteau S Laakkonen P Saksela O. Prolyl 4-Hydroxylase Subunit Alpha 1 (P4HA1) is a Biomarker of Poor Prognosis in Primary Melanomas, and its Depletion Inhibits Melanoma Cell Invasion and Disrupts Tumor Blood Vessel Walls. Mol Oncol (2020) 14(4):742–62. 10.1002/1878-0261.12649 PMC7138405 32053263

[30] Jeong H Lim KM Kim KH Cho Y Lee B Knowles BC. Loss of Rab25 Promotes the Development of Skin Squamous Cell Carcinoma Through the Dysregulation of Integrin Trafficking. J Pathol (2019) 249(2):227–40. 10.1002/path.5311 PMC8075409 31144312

[31] Chen J Liu A Lin Z Wang B Chai X Chen S. Downregulation of the Circadian Rhythm Regulator HLF Promotes Multiple-Organ Distant Metastases in non-Small Cell Lung Cancer Through PPAR/NF-kappab Signaling. Cancer Lett (2020) 482:56–71. 10.1016/j.canlet.2020.04.007 32289442

[32] Allotey J Fernandez-Felix BM Zamora J Moss N Bagary M Kelso A. Predicting Seizures in Pregnant Women With Epilepsy: Development and External Validation of a Prognostic Model. PloS Med (2019) 16(5):e1002802. 10.1371/journal.pmed.1002802 31083654 PMC6513048

[33] Han MZ Huang B Ni SL Wang J Li XG Bjerkvig R. A Validated Prognostic Nomogram for Patients With Newly Diagnosed Lower-Grade Gliomas in a Large-Scale Asian Cohort. Neuro Oncol (2020) 22(5):729–31. 10.1093/neuonc/noaa027 PMC7229241 32025722

[34] Wang SJ Lemieux A Kalpathy-Cramer J Ord CB Walker GV Fuller CD. Nomogram for Predicting the Benefit of Adjuvant Chemoradiotherapy for Resected Gallbladder Cancer. J Clin Oncol (2011) 29(35):4627–32. 10.1200/JCO.2010.33.8020 PMC3236647 22067404

[35] Stephenson AJ Scardino PT Eastham JA Bianco FJ Jr. Dotan ZA DiBlasio CJ. Postoperative Nomogram Predicting the 10-Year Probability of Prostate Cancer Recurrence After Radical Prostatectomy. J Clin Oncol (2005) 23(28):7005–12. 10.1200/JCO.2005.01.867 PMC2231088 16192588

[36] Ahn HK Lee S Park YH Sohn JH Jo JC Ahn JH. Prediction of Outcomes for Patients With Brain Parenchymal Metastases From Breast Cancer (BC): A New BC-specific Prognostic Model and a Nomogram. Neuro Oncol (2012) 14(8):1105–13. 10.1093/neuonc/nos137 PMC3408262 22693244

[37] Brandao M Caparica R Eiger D de Azambuja E. Biomarkers of Response and Resistance to PI3K Inhibitors in Estrogen Receptor-Positive Breast Cancer Patients and Combination Therapies Involving PI3K Inhibitors. Ann Oncol (2019) 30(Suppl_10):x27–42. 10.1093/annonc/mdz280 PMC6923785 31859350

[38] Zhou YQ Zhou Z Qian MF Gong T Wang JD. Association of Thyroid Carcinoma With Pregnancy: A Meta-Analysis. Mol Clin Oncol (2015) 3(2):341–6. 10.3892/mco.2014.472 PMC4360860 25798264

[39] Sag D Cekic C Wu R Linden J Hedrick CC. The Cholesterol Transporter ABCG1 Links Cholesterol Homeostasis and Tumour Immunity. Nat Commun (2015) 6:6354. 10.1038/ncomms7354 25724068 PMC4347884

[40] Ying H DePinho RA. Cancer Signaling: When Phosphorylation Meets Methylation. Cell Res (2014) 24(11):1282–3. 10.1038/cr.2014.103 PMC4220151 25104733

[41] Rao S Mondragon L Pranjic B Hanada T Stoll G Kocher T. AIF-Regulated Oxidative Phosphorylation Supports Lung Cancer Development. Cell Res (2019) 29(7):579–91. 10.1038/s41422-019-0181-4 PMC6796841 31133695

[42] Currie E Schulze A Zechner R Walther TC Farese RV Jr. Cellular Fatty Acid Metabolism and Cancer. Cell Metab (2013) 18(2):153–61. 10.1016/j.cmet.2013.05.017 PMC3742569 23791484

[43] Bery N Miller A Rabbitts T. A Potent KRAS Macromolecule Degrader Specifically Targeting Tumours With Mutant KRAS. Nat Commun (2020) 11(1):3233. 10.1038/s41467-020-17022-w 32591521 PMC7319959

[44] Savagner P. The Epithelial-Mesenchymal Transition (EMT) Phenomenon. Ann Oncol (2010) 21(Suppl 7):vii89–92. 10.1093/annonc/mdq292 20943648 PMC3379967

[45] Landi MT Bishop DT MacGregor S Machiela MJ Stratigos AJ Ghiorzo P. Genome-Wide Association Meta-Analyses Combining Multiple Risk Phenotypes Provide Insights Into the Genetic Architecture of Cutaneous Melanoma Susceptibility. Nat Genet (2020) 52(5):494–504. 10.1038/s41588-020-0611-8 32341527 PMC7255059

[46] Jiang H Zhou C Zhang Z Wang Q Wei H Shi W. Jagged1-Notch1-deployed Tumor Perivascular Niche Promotes Breast Cancer Stem Cell Phenotype Through Zeb1. Nat Commun (2020) 11(1):5129. 10.1038/s41467-020-18860-4 33046710 PMC7552407

[47] Senft E Lemound J Stucki-Koch A Gellrich NC Kreipe H Hussein K. Expression of Cyclin-Dependent Kinase Inhibitor 2A 16, Tumour Protein 53 and Epidermal Growth Factor Receptor in Salivary Gland Carcinomas is Not Associated With Oncogenic Virus Infection. Int J Oral Sci (2015) 7(1):18–22. 10.1038/ijos.2014.28 25012870 PMC4817540

